# THE EFFECT OF SHORT-TERM TREATMENT WITH BOTULINUM TOXIN A ON MUSCLE STIFFNESS IN STROKE PATIENTS: AN EXPLORATORY STUDY

**DOI:** 10.2340/jrm.v57.44318

**Published:** 2025-09-15

**Authors:** Jelena SIMIC, Kristin ØSTLIE, Fin BIERING-SØRENSEN, Bo BIERING-SØRENSEN, Derek John CURTIS, Arve OPHEIM

**Affiliations:** 1 Sunnaas Rehabilitation Hospital, Research Department, Norway; 2 Faculty of Medicine, University of Oslo, Oslo; 3 Innlandet Hospital Trust, Department of Physical Medicine and Rehabilitation, Ottestad, Norway; 4 Department of Clinical Medicine University of Copenhagen, Copenhagen; 5 Department of Brain and Spinal Cord Injuries, Bodil Eskesen Centre, Neuroscience Centre, Rigshospitalet, Copenhagen; 6 Neurological Department, Copenhagen University Hospital, Rigshospitalet, Copenhagen; 7 Department of Public Health, Faculty of Health and Medical Sciences, University of Copenhagen, Copenhagen; 8 Department of Pediatric Rehabilitation, Children’s Therapy Center, Copenhagen, Denmark; 9 Institute for Neuroscience and Physiology, University of Gothenburg, Sweden

**Keywords:** botulinum toxin type A, muscle stiffness, spasticity, stroke, shear wave elastography, muscle diagnostic imaging

## Abstract

**Objective:**

To examine whether muscle stiffness changes occur after botulinum toxin A treatment measured with shear wave elastography over a 3-month period and whether these changes are associated with walking function, active and passive ankle dorsiflexion, and participants’ goal achievements.

**Design:**

Prospective cohort study.

**Subjects/Patients:**

25 first stroke survivors with disabling spasticity and reduced walking function (mean age 52.6).

**Methods:**

Botulinum toxin A was administered in relevant calf muscles. Muscle stiffness measurements with shear wave elastography and clinical tests such as spasticity test (Modified Ashworth scale), maximal active and passive dorsiflexion of the ankle, and 10 m walk test were conducted at baseline, after 6 weeks, and after 3 months. Medial gastrocnemius muscle stiffness was measured in both the affected and the unaffected leg. The Goal Attainment Scale was used to evaluate therapy goal achievements.

**Results:**

Significantly reduced muscle stiffness was found at 6 weeks on the affected side, but not at 3 months, and no changes were present on the unaffected side. A moderate positive correlation between muscle stiffness and Goal Attainment Scale score was observed.

**Conclusion:**

Shear wave elastography could evaluate the effect of botulinum toxin A on muscle stiffness in stroke patients over a 3-month period. The correlation between muscle stiffness changes, goal attainment, and function need further longitudinal studies.

## LAY ABSTRACT

Spasticity is a condition that may occur after stroke and can cause further complications and decreased quality of life. Spasticity contributes to increased muscle stiffness. The objective of this study is to examine whether muscle stiffness changes after botulinum toxin A treatment and whether these changes correlate to clinical tests routinely used in clinical practice. Shear wave elastography is a type of ultrasound examination that measures the speed of wave propagation in muscle tissue. Greater muscle stiffness means higher propagation speed. We found that shear wave elastography can be used to monitor the effect of botulinum toxin therapy on the muscle stiffness, but long-term research is needed to investigate correlation with the clinical tests. Measuring muscle stiffness with shear wave elastography can help in the planning of treatment and in predicting the effect of the therapy and therefore contribute to better clinical practice for stroke patients with disabling spasticity.

Spasticity, a sensorimotor disorder characterized by intermittent or sustained involuntary muscle activation, is a common and potentially problematic consequence of upper motor neuron disorders such as stroke (1). A systematic review has shown that the incidence of post-stroke spasticity (PSS) was 39.5% (2) and a meta-analysis (3) found that 9.4% of stroke patients developed severe or disabling spasticity. PSS may lead to various complications such as pain, gait abnormalities, and reduced function, leading to difficulties in performing daily activities. Additionally, PSS can reduce health-related quality of life and increase the healthcare burden (4, 5).

The first-line treatment for focal, segmental, and multi-segmental spasticity is injection therapy with Botulinum Toxin A (BoNT-A) (6, 7). This treatment is widely recognized as a safe and well-established therapy for spasticity (6–8). BoNT-A is injected intramuscularly and acts by producing a cholinergic blockade at the neuromuscular junction. The clinical effect starts after 4–7 days and lasts for 12–20 weeks (6, 7). Due to reversibility of the effect, injections with BoNT-A have to be repeated in order to achieve the patients’ long-term goals (7, 8). Studies have shown that early treatment of spasticity with BoNT-A injections in stroke patients can prevent development of disabling spasticity in some patients (9, 10). However, there is still lack of documentation on whether BoNT-A treatment causes changes in muscle stiffness and muscle morphology (11), and whether these changes have an impact on walking function and achievement of therapy goals.

Shear wave elastography (SWE) is used to measure muscle stiffness (12, 13). This non-invasive sonographic method determines tissue stiffness by measuring the velocity of the ultrasound waves in the muscle tissue. SWE has been used to measure stiffness in plantar flexors (14–16) and biceps brachii (17, 18) among stroke patients, and SWE is considered feasible for use in clinical practice among patients with spasticity who receive anti-spasticity treatment (19–21). Also, SWE has been found to be a useful, supplementary method to assess spasticity in the medial gastrocnemius muscle (MGM) (20, 22). Reviews have suggested that SWE can be used to plan treatments, to identify muscles to be treated, and to evaluate the effect of BoNT-A injections in individuals with stroke. The correlation between clinical assessments and SWE is also described as promising (23, 24). However, methodological issues such as selection of target muscles, transducer position, and SWE settings as well as the lack of a standardized measurement protocol may affect the reliability of this method (23, 25–27).

The aim of this study was to examine whether changes in muscle stiffness occur after BoNT-A treatment over a 3-month period and whether these changes measured with SWE were associated with changes in walking function, active and passive ankle dorsiflexion, and the participant’s goal achievements related to walking function.

## METHODS

### Design

We performed a prospective cohort study. Participants were consecutively recruited from the Spasticity Clinic, Sunnaas Rehabilitation Hospital, Norway from 30 August 2022 to 27 August 2024. All patients with a single stroke, both acute (< 3 months post-stroke) and chronic (> 3 months post-stroke) patients were included. The participants had increased stiffness (Modified Ashworth Scale [MAS] greater than 0) in 1 or more of the calf muscles in the affected leg, and disabling spasticity (decreased joint range of motion, mobility impairment, or symptoms related to muscle stiffness) established during clinical assessment (8).

Persons excluded from the study were those who had had more than 1 stroke, who suffered from other neurological conditions affecting lower limb muscle function, who were unable to cooperate, or could not be positioned safely and/or comfortably for the measurements.

### Treatment procedure

BoNT-A injections were administered in relevant spastic leg muscles, including the medial gastrocnemius muscle (MGM), according to recommended guidelines, using ultrasound-guided technique and electromyography simultaneously (28, 29). All injections were performed by an experienced physician (JS). The doses injected into the MGM were selected by the administering physician based on clinical tests and prior experience. The doses ranged from 50 AE to 100 units, with a median of 70 units.

### Outcome measurements

*Shear Wave Elastography (SWE).* SWE was used to measure muscle stiffness in MGM before and after BoNT-A injection treatment. Muscle stiffness is expressed as shear wave velocity (m/s). The measurements were performed at baseline, after 6 weeks, and after 3 months by the same physician as administered the injections.

SWE measurements were performed with the participant in the supine position with the knee extended. As the aim was to examine the effect of BoNT-A injection treatment on muscle stiffness, a position in which the muscle was maximally stretched – i.e., with the ankle joint in maximal dorsiflexion (max DF) – was chosen. In addition to this, the position of 30° plantar flexion (30° PF) was chosen in order to have a common joint angle across all participants. The ankle joint was held at 30° PF using a custom-made orthosis, while at max DF it was held manually, every time by the same person to minimize variation. Muscle stiffness was measured in MGM in both the affected and the unaffected leg, to allow for control with an un-injected muscle, and to evaluate treatment effects vs muscle changes due to stroke itself (30), inactivity, or other patient-related factors.

**Fig. 1:**
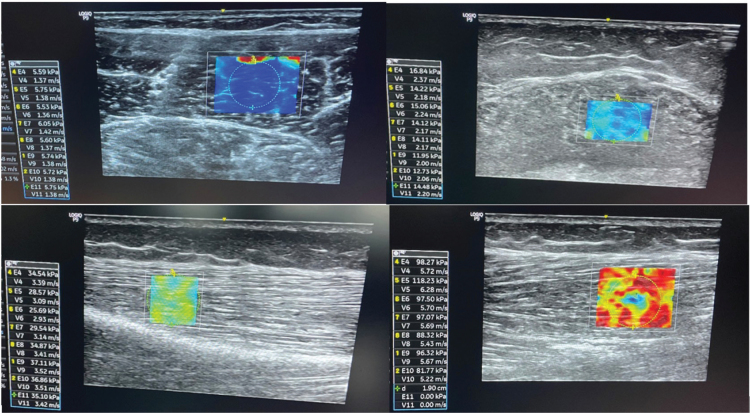
The shear wave elastography colour scale. The shear wave elastography colour scale ranges from lower velocities/muscle stiffness (1–2 m/s, dark blue) to higher velocities/muscle stiffness (> 4 m/s, dark red).

The muscles were scanned using SWE with a linear array transducer and musculoskeletal preset (LOGIQ P9 R3 with L3-12-RS probe, GE Healthcare, Chicago, IL, USA). The SWE device shows real-time colour-coded images with a frame rate of 1 Hz in a pre-defined region of interest (ROI). The SWE colour scale shows muscle stiffness measured using ultrasound propagation velocity. A higher velocity of ultrasound propagation indicates greater muscle stiffness (Fig. 1).

The muscles were scanned in the direction of the muscle fibres, with the transducer oriented parallel to the fascicles at the mid-belly region of each muscle. The distance from the probe to the medial malleolus was recorded to minimize variation between measurements. Transmission gel was used to ensure even contact and low pressure on the muscle. All participants were instructed to remain as relaxed as possible throughout the testing. In post-processing, a circular ROI (approx. diameter 3–4 cm) was used to cover as large a part of the muscle as possible. The mean sound wave velocity (m/s) over a 10 s period (10 frames) was extracted and used in the analysis.

*Clinical tests.* The following clinical tests were carried out at baseline, and after 6 weeks and 3 months:

- Assessment of spasticity in the calf muscles with MAS (1–4, included 1+) (4, 30).
- Maximal active and passive dorsiflexion of the ankle assessed using a goniometer (31).
- Walking function was measured with the 10 meter walk test (32). The walking speed was measured in m/s. The test provides information on functional mobility, gait, and vestibular function. It has demonstrated excellent reliability and validity in persons with spastic paresis (32). All tests were performed by 2 experienced physiotherapists.

Prior to injection with BoNT-A, the participants defined their treatment goals related to walking function and outcome expectations together with the treating physician using the Goal Attainment Scale (GAS). The same scale was used to evaluate the achievement of the therapy goals after 6 weeks and 3 months. The GAS is a method used to measure the achievement of a participant’s individual goals (33). Each goal is rated on a 5-point scale: the expected achievement level is scored at 0, whereas a better than expected outcome is scored at +1 (somewhat better) or +2 (much better); less than expected outcomes are scored at –1 (somewhat less) and –2 (much less).

### Statistical analyses

Statistical analyses were conducted using Python (www.Python.org) and Python’s Pandas package (https://pandas.pydata.org/) for data manipulation, NumPy for numerical computations (-https://numpy.org/), SciPy for statistical testing (https://scipy.org/), and Matplotlib (https://matplotlib.org/) and Seaborn (https://seaborn.pydata.org/) for data visualization. The Shapiro–Wilk test was used to assess the normality of the collected variables. As there were no normally distributed paired data, the non-parametric Wilcoxon signed-rank test was applied to analyse statistical significance at the group level. Statistical significance was considered at a threshold of *p* < 0.05. The results of the Botulinum Toxin A treatment effect were presented at both group and individual level as notched box and waterfall charts, respectively.

To explore associations between variables, Spearman’s rank correlation coefficient (ρ) was used for monotonic, but potentially nonlinear associations. For correlation analyses, the correlation coefficient and the 95% confidence interval (CI) were calculated.

## RESULTS

Initially, there were 27 participants, 2 of whom were excluded after 6 weeks. One was excluded due to difficulties with the follow-up schedule related to the study and the other decided to withdraw for personal reasons. Seventeen participants had prior BoNT-A treatment of the calf muscles, 8 were treatment naive. The time since last BoNT-A treatment was minimum 3 months and maximum 7.5 months. Six participants were on oral medication for spasticity and all of them used Lioresal (baclofen). Nineteen participants were using oral medications that could cause increased muscle stiffness as a side effect such as statins. Nineteen participants had physiotherapist-guided training and 6 did not. Characteristics of the study sample are presented in Table I.

**Table I:** Characteristics of the study group

Variable	Details	
Total participants, *n*	25	
Mean age, (SD)	52.6 (9.8)	
Age range (years)	26 to 70	
Sex	Female: 6	Male: 19
Cause of stroke	Haemorrhage: 7	Thrombosis: 18
Prior BoNT-A treatment in calf muscles	Yes: 17	No: 8
Additional medication for spasticity	Yes: 6	No: 19
Medication impacting muscle stiffness	Yes: 19	No: 6
Use of walking aids	Yes: 14	No: 11
Follow-up by community physiotherapists	Yes:19	No: 6

On the affected side injected with BoNT-A, there were significant reductions in muscle stiffness in the MGM from baseline to 6 weeks in both ankle joint positions (Table II). From 6 weeks to 3 months there was a significant increase in muscle stiffness in both positions. No significant changes were found from baseline to 3 months in either position. There were no significant differences between those stroke patients who had received treatment with BoNT-A before being included in the study and those who had not.

**Table II:** Median muscle stiffness in the medial gastrocnemius muscle on the affected and unaffected side measured in maximal dorsiflexion and 30^o^ plantar flexion using shear wave elastography

Item	Baseline *n* = 25	6 weeks *n* = 25	*p*-value^a^ baseline–6 weeks	3 months *n* = 25	*p*-value^a^ 6 weeks–3 months	*p*-value^a^ baseline–3 months
SW velocity (m/s) affected side, max DF (IQR)	3.23 (1.59)	2.48 (0.8)	0.002	2.66 (0.94)	0.048	0.411
SW velocity (m/s) affected side, 30°PF (IQR)	1.79 (0.89)	1.55 (0.91)	0.031	1.65 (0.64)	0.015	0.339
SW velocity (m/s) unaffected side, max DF (IQR)	2.45 (0.69)	2.20 (0.90)	0.090	2.30 (0.57)	0.427	0.178
SW velocity (m/s) unaffected side, 30°PF (IQR)	1.61 (0.63)	1.52 (0.68)	0.338	1.43 (0.42)	0.076	0.153

a The Wilcoxon signed-rank test.

SW: shear wave: DF: dorsiflexion; PF: plantarflexion; IQR: interquartile range.

On the non-affected side, there were no significant changes in muscle stiffness from baseline to 6 weeks, nor from baseline to 3 months, in either position.

**Fig. 2:**
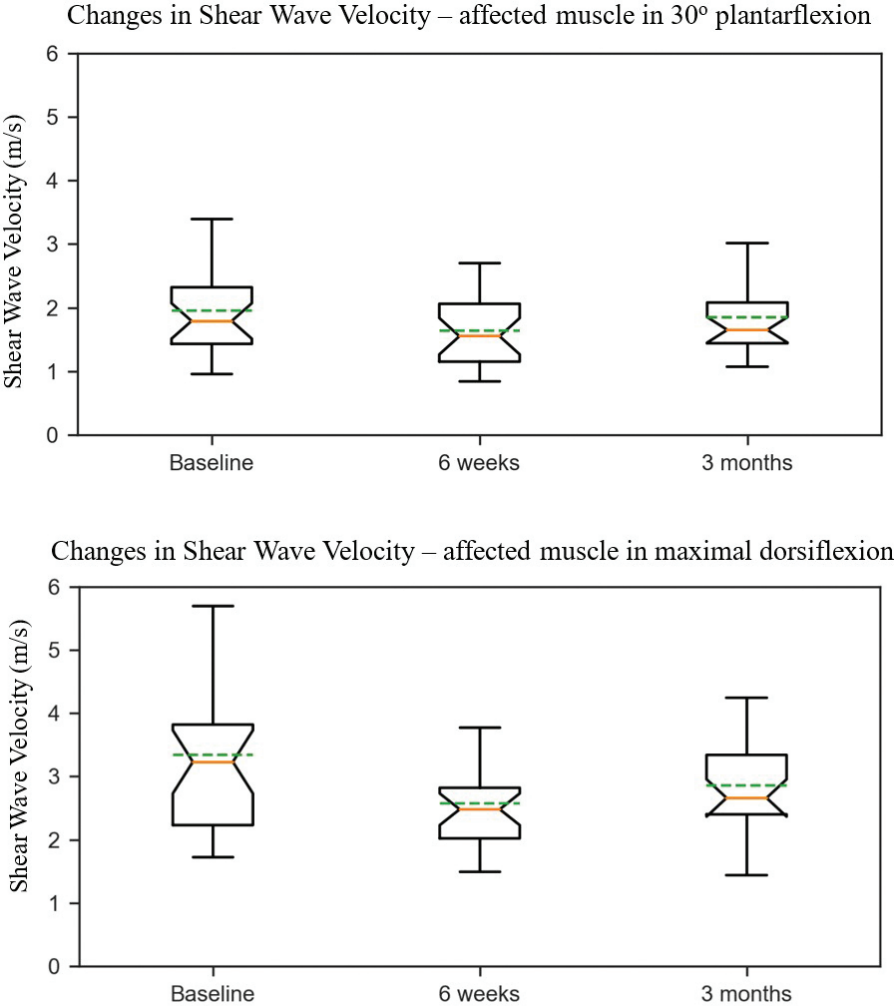
Notched box plot illustrating shear wave velocity in medial gastrocnemius muscle at baseline, 6 weeks, and 3 months after Botulinum Toxin-A injections in 30^o^ plantarflexion and maximal dorsiflexion.

Fig. 2 shows the changes in muscle stiffness as notched box plots: the red line is the median, the box presents the IQR, while the notched part present 95% CI of the median. Non-overlapping of notches between baseline and 6 weeks indicates a statistically significant reduction of muscle stiffness. The muscle stiffness was lowest after 6 weeks in both positions with less variation in max DF.

**Fig. 3:**
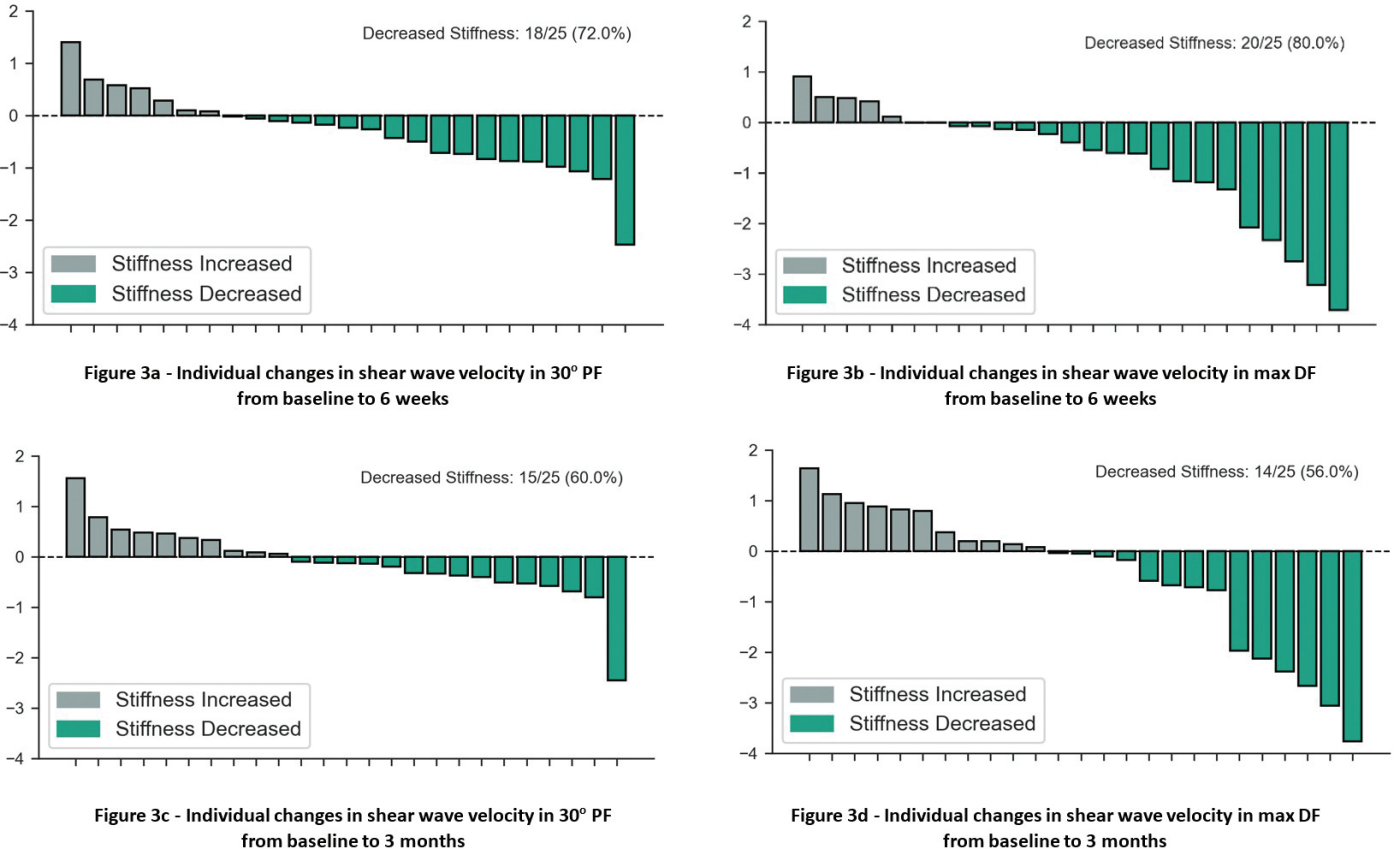
Waterfall plots presenting individual changes in shear wave velocity/muscle stiffness in the affected medial gastrocnemius muscle after Botulinum Toxin-A therapy in 30^o^ plantar flexion (30^o^ PF) and maximal dorsiflexion (max DF) (A and B) from baseline to 6 weeks and (C and D) from baseline to 3 months.

Fig. 3 illustrate the changes on an individual level, i.e., changes occurring in individual participants after 6 weeks and after 3 months. Most participants (72% in 30° PF and 80% in max DF) experienced decreased muscle stiffness after 6 weeks (Fig. 3A and B). Fig. 3C and 3D show that 60% (15 of 18) of participants in 30° PF and 56% (14 of 20) in max DF still had less muscle stiffness measured with SWE after 3 months than at baseline.

A moderate positive correlation was seen between changes in muscle stiffness from baseline to 3 months in 30^o^ PF and goal attainment (GAS score) (ρ = 0.400, *p* = 0.05). No other significant correlations were observed between changes in muscle stiffness and either walking function (10 m walk test) or GAS score at 30^o^ PF (Table SI).

We did not find any significant correlations between changes in muscle stiffness from baseline to 3 months and changes in walking function or GAS score in max DF (Table SII).

We did not find significant changes in passive or active ankle joint movement from baseline to 6 weeks or from baseline to 3 months, or any significant correlation between changes in joint movement and changes in muscle stiffness.

## DISCUSSION

In our study, we found significant changes in muscle stiffness in the MGM on the affected side from baseline to 6 weeks after BoNT-A treatment, measured in both positions of the ankle, but no significant changes from baseline to 3 months. No significant changes were found in the non-affected leg.

Our findings are in line with the well-known effect cycle of BoNT-A injection treatment and prior studies’ recommendations for evaluation of the treatment. These studies have shown that the best effect of BoNT-A injections occurs after 4 weeks; however, they recommend evaluation of the active therapy goals after 6 weeks due to need for training after treatment to achieve maximal effect (34, 35).

Furthermore, our findings suggest that SWE can be used to evaluate the effect of injection treatment with BoNT-A on muscle stiffness in patients with PSS. This is in line with prior studies showing that SWE may be a reliable method to evaluate muscle stiffness after stroke (16, 18, 19, 26) as well as to evaluate the effect of BoNT-A treatment in spastic muscles in persons with stroke (19, 20, 23, 24). A study has shown that increased SW velocity can be associated with the results of spasticity tests such as MAS (14).

In participants who had decreased muscle stiffness in the MGM at the 6 weeks evaluation in the affected leg, we observed a slight stiffness increase at 3 months’ follow-up, without the stiffness rising to the baseline levels for all individuals. This might suggest that repeated BoNT-A injections can cause a gradual decrease in muscle stiffness over time. This is a finding with high clinical relevance and needs further investigation with longitudinal study designs. Several studies have examined the effect of BoNT-A therapy with SWE after 2–4 weeks, which may be considered a short time after injection (14, 17, 20), or included a small number of participants (16, 18, 19), which may have an impact on their findings. To date, it is therefore still unknown whether changes in muscle stiffness after BoNT-A treatment have a lasting effect.

In addition to this, there is still a lack of knowledge as to whether muscle stiffness measurements with SWE impact on clinical decisions such as the choice of muscles to be injected or doses applied, as well as to whether SWE can be used to predict the effect of injection therapy with BoNT-A.

We found only a moderate positive correlation between changes in muscle stiffness and GAS results in the period from baseline to 3 months in 30° PF. This could be explained either by the fact that the patients’ goals were not uniform or that some of them were unrealistic. In addition to this, the observation period may have been too short. Many factors such as age, use of medications, physical activity, stretching of muscles, and others may have an impact on both muscle stiffness and goal attainment. We believe that, to better understand this relationship, future studies should involve participants with similar therapy goals. Also, participants should preferably be observed over a longer period.

We did not find any correlations between changes in muscle stiffness and walking function, presumably because a significant number of participants had poor walking function at baseline. In addition to this, there were possibly many other factors, not only spasticity, which may have had an impact on walking function, such as lack of coordination, reduced balance, reduced sensibility, paresis, contractures, and physiotherapist-guided training. We believe that observation over a longer period is needed.

SWE measurements can be changed due to muscle length and position and therefore prior studies emphasize the need for a standardized measurement protocol (22, 25, 26). Our study is in line with this, because we found different results in ankle positions on group and individual level. We measured muscle stiffness with SWE in 2 ankle positions, 30^o^ PF and max DF. The results that show effectiveness of BoNT-A therapy on muscle stiffness were greater in max DF at a group level. However, at an individual level the results were greater in 30^o^ PF.

### Limitations

Limitations of this study are small sample size, large variations in the patients’ conditions such as the level of spasticity severity, time after stroke, age, different levels of activity and training, lack of initiative due to mild cognitive impairment, different individual goals related to walking function, as well as other neurological impairments related to stroke that may have an impact on walking function. These variations reduce the possibilities to draw unambiguous conclusions from this study. Furthermore, SWE as a method to measure muscle stiffness has some limitations. One of the disadvantages with SWE measurements is that this measurement cannot distinguish between neural and non-neural changes and includes both spasticity and connective tissue stiffness in response to passive stretch (36). However, our findings show that SWE-measured muscle stiffness changed only in injected muscles, suggesting that this measurement, in addition to other clinical tests, may contribute to distinguishing these 2 components. This is a suggested topic for further research.

Also, SWE results depend on tissue density and shear wave velocity. In our study, we assume shear wave velocity to be highly correlated with muscle stiffness and compared muscle stiffness in the same participant at different points in time. We did not compare muscle stiffness between different participants. Thus, the fact that muscle density differs from patient to patient (37, 38) should not be a concern in our study. It is, however, possible that muscle density changed in some patients over time, which could have affected the calculated shear modulus, i.e., muscle stiffness at different points in time. In future studies, it would be interesting to investigate if, and how, muscle density changes over time in the stroke population.

A potential limitation of our study is also the lack of blinding, i.e., the fact that the treatment and its evaluation have been performed by the same physician, which may have influenced the results and made them subject to bias.

### Conclusion

We found that BoNT-A injections reduced muscle stiffness measured with SWE after 6 weeks in individuals with stroke. In addition, our results demonstrated that the median level of muscle stiffness did not fully return to the baseline levels 3 months after therapy, which provides a valuable direction for further and longitudinal research on the effect of long-term BoNT-A therapy. A moderate positive correlation between muscle stiffness and GAS score was observed. The correlation between changes in muscle stiffness, goal attainments, and function need to be explored further in longitudinal studies.

## Supplementary Material



## Trial details

This was a prospective cohort study investigating the effect of Botulinum Toxin A on muscle stiffness in patients with first stroke. The study was conducted at Sunnaas Rehabilitation Hospital, Norway between August 2022 and August 2024.
