# Supplementary material for: THE EFFECT OF SHORT-TERM TREATMENT WITH BOTULINUM TOXIN A ON MUSCLE STIFFNESS IN STROKE PATIENTS: AN EXPLORATORY STUDY
Source: J Rehabil Med. 2025 Sep 15;57:44318. doi: 10.2340/jrm.v57.44318 (PMC12447518; doi:10.2340/jrm.v57.44318)
Supplement: Supplementary file 1 [file JRM-57-44318-s1.pdf]

Supplementary material has been published as submitted. It has not been copyedited, or typeset by Journal of Rehabilitation Medicine

Table SI: Spearman Correlations between changes in muscle stiffness and changes in walking function in 30º plantar flexion and maximal dorsiflexion for 25 participants.

Time Interval	Position	Median Δ Stiffness	Median Δ Walk Speed (m/s)	ρ (95% CI)	P
Baseline to 6 Weeks	Maximal Dorsiflexion	-0.39	0.02	0.22 (-0.25 to 0.64)	0.30
	30° Plantarflexion	-0.24		0.11 (-0.34 to 0.51)	0.61
Baseline to 3 Months	Maximal Dorsiflexion	-0.05	0.04	0.09 (-0.28 to 0.46)	0.68
	30° Plantarflexion	-0.13		0.26 (-0.03 to 0.60)	0.22
6 Weeks to 3 Months	Maximal Dorsiflexion	0.34	-0.01	-0.24 (-0.53 to 0.14)	0.27
	30° Plantarflexion	0.29		-0.27 (-0.62 to 0.27)	0.20

Table SII: Spearman Correlations between changes in muscle stiffness and changes in Goal Attainment Scale (GAS) results in 30º plantar flexion and maximal dorsiflexion for 25 participants.

Time Interval	Position	Median Δ Stiffness	Median GAS	ρ (95% CI)	P
Baseline to 6 Weeks	Maximal Dorsiflexion	-0.39	0	0.04 (-0.35 to 0.39)	0.84
	30° Plantarflexion	-0.24		0.03 (-0.35 to 0.35)	0.89
Baseline to 3 Months	Maximal Dorsiflexion	-0.05	0	0.08 (-0.33 to 0.47)	0.72
	30° Plantarflexion	-0.13		0.40 (0.02 to 0.71)	0.05
6 Weeks to 3 Months	Maximal Dorsiflexion	0.34	0	-0.12 (-0.48 to 0.27)	0.56
	30° Plantarflexion	0.29		0.06 (-0.38 to 0.49)	0.76
